# Accurate prediction of drug-protein interactions by maintaining the original topological relationships among embeddings

**DOI:** 10.1186/s12915-025-02338-0

**Published:** 2025-08-05

**Authors:** Yanfei Li, Xiran Chen, Shuqin Wang, Jinmao Wei

**Affiliations:** 1 College of Computer Science, Nankai University, 300071 Tianjin, China; 2 College of Computer and Information Engineering, Tianjin Normal University, 300387 Tianjin, China; 3 National Heart & Lung Institute, Imperial College London, SW3 6LY London, UK

**Keywords:** Drug-protein interaction, Topological relationships, Deep neural network, Guilt-by-association, Prior loss function

## Abstract

**Background:**

Learning-based methods have recently demonstrated strong potential in predicting drug-protein interactions (DPIs). However, existing approaches often fail to achieve accurate predictions on real-world imbalanced datasets while maintaining high generalizability and scalability, limiting their practical applicability.

**Results:**

This study proposes a highly generalized model, GLDPI, aimed at improving prediction accuracy in imbalanced scenarios by preserving the topological relationships among initial molecular representations in the embedding space. Specifically, GLDPI employs dedicated encoders to transform one-dimensional sequence information of drugs and proteins into embedding representations and efficiently calculates the likelihood of DPIs using cosine similarity. Additionally, we introduce a prior loss function based on the guilt-by-association principle to ensure that the topology of the embedding space aligns with the structure of the initial drug-protein network. This design enables GLDPI to effectively capture network relationships and key features of molecular interactions, thereby significantly enhancing predictive performance.

**Conclusions:**

Experimental results highlight GLDPI’s superior performance on multiple highly imbalanced benchmark datasets, achieving over a 100% improvement in the AUPR metric compared to state-of-the-art methods. Additionally, GLDPI demonstrates exceptional generalization capabilities in cold-start experiments, excelling in predicting novel drug-protein interactions. Furthermore, the model exhibits remarkable scalability, efficiently inferring approximately $1.2 \times 10^{10}$ drug-protein pairs in less than 10 h.

**Supplementary Information:**

The online version contains supplementary material available at 10.1186/s12915-025-02338-0.

## Background

In drug development, it is crucial to determine which proteins a drug can interact with [1, 2]. While wet-lab experiments are considered reliable for the identification of drug-protein interactions (DPIs), they are associated with significant drawbacks, including time-consuming procedures and high costs, which hinder their scalability [3, 4]. On the contrary, computational methods are efficient and cost-effective. Moreover, they can provide reliable candidates for subsequent wet lab experiments and offer valuable insights into molecular interaction processes.

The computational methods of DPI prediction can be roughly divided into network-based and learning-based methods. Due to demonstrating relatively accurate predictive performance, network-based techniques such as random walks and graph regularization [5, 6] have been applied in DPI prediction. The foundation of network-based methods is the guilt-by-association principle, which assumes that similar drugs will interact with similar proteins [1]. Network-based methods achieve information updating by constructing drug-protein heterogeneous networks and propagating interaction information to similar neighboring nodes on the network [7–9]. However, these methods primarily focus on molecular-level comparisons of drugs and proteins, neglecting the topological information inside the drugs and proteins [10]. Therefore, they fail to capture critical features contributing to molecular binding and struggle to learn complex interactions between drugs and proteins. Moreover, the reliance on network information typically results in poor scalability and limited generalization ability for such methods.

In recent years, the rapid advancement of deep learning has driven the widespread adoption of data-driven, highly generalizable methods for DPI prediction. These approaches encode drugs and proteins into high-dimensional feature vectors and utilize neural networks to automatically learn molecular representations and interaction patterns, thereby enhancing predictive performance. Researchers have explored various deep-learning architectures for this task. DeepDTA [11], DeepCompoundNet [12], DeepConv-DTI [13], and DeepDTAF [14] apply convolutional neural networks to extract informative features from drug and protein sequences. SGCL-DTI [10], SMGCL [15], CCL-ASPS [16], and PSC-CPI [17] enhance feature representation learning through contrastive learning techniques, constructing positive and negative sample pairs to improve model discrimination. MCANet [18], DrugBAN [19], HyperAttentionDTI [20], and BACPI [21] utilize attention mechanisms to dynamically focus on critical interaction information, thereby enhancing interpretability. Meanwhile, BridgeDTI [4], MvGraphDTA [22], and CSCo-DTA [23] use graph neural networks to model the topological structures of drugs and proteins, capturing complex molecular interactions. Once these models extract meaningful feature representations, they typically rely on fully connected networks to further model drug-target interactions. However, challenges such as class imbalance often hinder their effectiveness. In DPI datasets, the imbalance is extreme, with positive samples (known DPIs) accounting for less than 0.1% of the total [24, 25], vastly outnumbered by negative samples (unknown DPIs). This imbalance significantly impacts the predictive accuracy of deep learning models, which tend to perform well in balanced settings but struggle in real-world imbalanced scenarios. To mitigate overfitting and accelerate training, existing methods often construct balanced datasets by randomly selecting an equal number of negative samples to match the positive samples for training and evaluation [14, 26]. However, these approaches bias the model’s understanding of the data distribution, leading to substantial performance degradation when applied to imbalanced real-world data. To address this limitation, previous studies have explored techniques such as increasing the proportion of negative samples in the training set [27, 28] and adjusting class weights during training [18]. Despite these efforts, balancing the trade-off between enhancing model generalizability and avoiding overfitting remains a significant challenge.

This study proposes a topology-maintaining molecular embedding method that effectively addresses the aforementioned challenges at the algorithmic level. The technique not only inherits the high generalizability advantages of embedding-based learning approaches but also measures the likelihood of molecular reactions through the distances between embeddings, thereby achieving linear time complexity. Compared to the quadratic time complexity commonly found in existing methods, this approach significantly improves computational efficiency and scalability. Furthermore, by ensuring that the topological structure of molecular embeddings aligns with the relationships in the drug-protein heterogeneous network, the model can effectively capture key topological information between molecules. As a result, drugs and proteins that are structurally or functionally similar to known interactions are more likely to form new interactions. This design inherently adheres to the “Guilt-by-Association” principle, where similar entities tend to participate in similar interactions. Unlike traditional learning-based methods, the guilt-by-association principle operates independently of training data [29], making it robust to biased data distributions. By leveraging this mechanism, the proposed method excels in highly imbalanced scenarios, effectively identifying potential drug-protein interactions. The preservation of topological relationships enables the model to comprehensively understand DPI, even when trained on small or imbalanced datasets. Therefore, the proposed method demonstrates high accuracy in predicting drug-protein interactions, particularly in cases of severe data imbalance, overcoming the challenges faced by traditional deep learning methods.

Specifically, we present GLDPI, a novel deep-learning approach for predicting drug-protein interactions (DPIs). GLDPI employs a fully connected encoding network to map drug and protein features into a shared embedding space, where interaction likelihoods are assessed using cosine similarity. To enhance predictive power, a heterogeneous drug-protein network integrating drug similarity, protein similarity, and known DPIs is constructed. A prior loss function, based on the guilt-by-association principle, ensures alignment between molecular embeddings and the network’s topological structure. By leveraging both network-level structural associations and atom-level interaction features, GLDPI effectively captures comprehensive knowledge, even when trained on small-scale or imbalanced datasets. This design enables GLDPI to achieve strong predictive performance, high generalizability, and scalability, particularly in scenarios with severe class imbalances. Experimental results demonstrate that GLDPI significantly outperforms state-of-the-art methods on highly imbalanced benchmark datasets. In cold-start experiments, GLDPI excels in predicting novel drug-protein interactions, achieving over 30% improvements in AUROC and AUPR compared to existing approaches. Furthermore, the model exhibits exceptional inference efficiency, predicting tens of billions of drug-protein pairs in under 10 h.

## Results and discussion

### Evaluation and metrics

We evaluated GLDPI using two benchmark datasets: BioSNAP [30] and BindingDB [31, 32]. The balanced BioSNAP dataset contains 27,454 interactions involving 4510 drugs and 2181 proteins, while the balanced BindingDB dataset includes 49,199 interactions involving 14,653 drugs and 2623 proteins. Following previous studies [3, 18, 32], both datasets were divided into training, validation, and test sets in a 7:1:2 ratio. A 1:1 negative sampling strategy was employed during training, where each positive drug-protein pair was randomly matched with a negative one. Although false negative samples are an inherent challenge in DPI prediction, prior work has shown that their occurrence in randomly sampled unlabeled data is typically below 0.1% [24, 25], making most sampled negatives reasonably reliable. To further mitigate the potential impact of false negatives, multiple rounds of negative sampling followed by averaging can be considered. Statistically, this helps reduce the variance introduced by any single sampling process, resulting in more stable and generalizable model performance. Additionally, to evaluate performance under imbalanced conditions, we extended the test set with randomly selected negative samples from unseen drug-protein pairs, constructing test scenarios with positive-to-negative ratios of 1:10, 1:100, and 1:1000.

We compared GLDPI with five state-of-the-art baselines in a variety of settings: MolTrans [3], DeepConv-DTI [13], ConPLex [2], MCANet [18], DrugBAN [19]. The area under the receiver operating characteristic curve (AUROC), the area under the precision versus recall curve (AUPR), Accuracy and F1_score are used as evaluation metrics. When dealing with imbalanced datasets, AUPR is often considered a better evaluation metric than AUROC. AUROC is insensitive to the sample distribution in imbalanced datasets, and samples from the majority class may dominate its calculation. In contrast, AUPR pays more attention to the classification performance of the minority class, making it more reliable and accurately reflecting the predictive ability of the model when dealing with imbalanced datasets.

### Implementation

We implemented GLDPI using PyTorch 1.12.0 and the Adam optimizer. The Morgan fingerprint dimension $d_m$ and protein feature dimension $d_t$ were set as 1024 and 1280, respectively. Hyperparameters were optimized via grid search, with a learning rate of 0.00001 and a maximum of 2000 iterations. Both drug and protein encoders used fully connected networks with layer sizes [2048, 512]. Key hyperparameters were set as $\lambda =1/3$ and $t=3$. Baseline models were configured following the parameter settings recommended in their respective papers.

### Prediction performance

To validate the predictive performance of the models, we trained GLDPI and baseline methods on balanced datasets. In addition to evaluating a 1:1 balanced test set, we also augmented the negative samples in the test set to construct three imbalanced test sets with ratios of 1:10, 1:100, and 1:1000, respectively. The experimental results are presented in Tables 1, 2, 3, and 4 (see Additional file 1: Table S1 and S2 for further statistics). As shown in Table 1, across the four different test scenarios for both datasets, GLDPI consistently achieved the highest AUROC scores, particularly outperforming all baseline methods in severely imbalanced test sets. As the proportion of negative samples increased, the performance of baseline methods tended to decline, while GLDPI exhibited minimal fluctuation, demonstrating strong robustness against noise introduced by negative samples. GLDPI leverages the GBA_Loss mechanism to maintain the topological relationships between embeddings, enabling it to capture richer structured knowledge in the drug-protein interaction network. Compared to traditional methods, GLDPI emphasizes the overall network relationships rather than the characteristics of individual data points, making it less sensitive to variations in the proportion of negative samples.

**Table 1:** The performance of models on the AUROC metric under different imbalance ratios (**best**, *second best*)

Method	1:1	1:10	1:100	1:1000
BioSNAP				
MolTrans	0.881	0.881	0.881	0.881
DeepConv-DTI	0.904	0.904	0.904	0.904
ConPLex	0.891	0.892	0.892	0.892
MCANet	*0.922*	*0.922*	*0.923*	*0.923*
DrugBAN	0.912	0.911	0.911	0.911
** GLDPI**	**0.958**	**0.957**	**0.957**	**0.957**
BindingDB				
MolTrans	0.929	0.756	0.731	0.728
DeepConv-DTI	0.964	0.850	0.833	0.831
ConPLex	0.888	0.857	*0.852*	*0.852*
MCANet	0.960	0.834	0.816	0.814
DrugBAN	*0.964*	*0.862*	0.846	0.844
** GLDPI**	**0.970**	**0.968**	**0.968**	**0.968**

**Table 2:** The performance of models on the AUPR metric under different imbalance ratios (**best**, *second best*)

Method	1:1	1:10	1:100	1:1000
BioSNAP				
MolTrans	0.890	0.555	0.169	0.026
DeepConv-DTI	0.905	0.564	0.137	0.016
ConPLex	0.911	*0.698*	*0.389*	*0.123*
MCANet	0.916	0.589	0.147	0.018
DrugBAN	*0.916*	0.605	0.170	0.021
** GLDPI**	**0.968**	**0.862**	**0.627**	**0.274**
BindingDB				
MolTrans	0.903	0.216	0.025	0.003
DeepConv-DTI	0.953	0.349	0.048	0.005
ConPLex	0.871	*0.696*	*0.467*	*0.177*
MCANet	0.946	0.327	0.044	0.005
DrugBAN	*0.953*	0.419	0.065	0.007
** GLDPI**	**0.961**	**0.919**	**0.827**	**0.557**

**Table 3:** The performance of models on the Accuracy metric under different imbalance ratios (**best**, *second best*)

Method	1:1	1:10	1:100	1:1000
BioSNAP				
MolTrans	0.809	0.913	0.985	0.998
DeepConv-DTI	0.837	0.921	0.975	0.991
ConPLex	0.829	*0.943*	*0.990*	*0.998*
MCANet	*0.855*	0.927	0.974	0.979
DrugBAN	0.839	0.927	0.983	0.993
** GLDPI**	**0.908**	**0.967**	**0.993**	**0.999**
BindingDB				
MolTrans	0.869	0.796	0.966	0.996
DeepConv-DTI	*0.916*	0.874	0.952	0.963
ConPLex	0.839	*0.952*	*0.992*	*0.998*
MCANet	0.906	0.847	0.967	0.994
DrugBAN	0.907	0.891	0.959	0.981
** GLDPI**	**0.919**	**0.980**	**0.996**	**0.999**

**Table 4:** The performance of models on the F1_score metric under different imbalance ratios (**best**, *second best*)

Method	1:1	1:10	1:100	1:1000
BioSNAP				
MolTrans	0.811	0.533	0.251	0.091
DeepConv-DTI	0.838	0.601	0.245	0.046
ConPLex	0.822	*0.661*	*0.443*	*0.200*
MCANet	0.857	0.631	0.283	0.046
DrugBAN	*0.843*	0.619	0.290	0.065
** GLDPI**	**0.910**	**0.810**	**0.620**	**0.330**
BindingDB				
MolTrans	0.834	0.281	0.059	0.011
DeepConv-DTI	*0.898*	0.458	0.112	0.014
ConPLex	0.804	*0.693*	*0.546*	*0.314*
MCANet	0.889	0.409	0.097	0.015
DrugBAN	0.888	0.495	0.142	0.021
** GLDPI**	**0.902**	**0.887**	**0.812**	**0.557**

Table 2 compares the performance of various models on the AUPR metric under different imbalance ratios, revealing GLDPI’s consistent superiority across all scenarios on both the BioSNAP and BindingDB datasets. This advantage is particularly pronounced in highly imbalanced settings, such as 1:100 and 1:1000. For instance, in the 1:1000 test set of the BioSNAP dataset, GLDPI achieved an AUPR of 0.274, significantly outperforming the next-best score of 0.123. Similarly, on the BindingDB dataset, GLDPI reached an AUPR of 0.557, nearly three times higher than the second-best result of 0.177 from ConPLex. Beyond AUPR, GLDPI also demonstrates exceptional accuracy and F1-score performance under various imbalance ratios. As shown in Table 3, it achieves the highest accuracy across both datasets, maintaining robustness even under severe imbalance (e.g., 0.999 on BioSNAP and BindingDB in the 1:1000 ratio). Similarly, Table 4 highlights GLDPI’s superior F1-scores, particularly under extreme imbalances. For example, at a 1:1000 ratio, GLDPI achieves an F1-score of 0.330 on BioSNAP and 0.557 on BindingDB, surpassing other models whose performance drops significantly in these scenarios. These results underscore GLDPI’s effectiveness in addressing class imbalance, capturing meaningful information from limited positive samples, and maintaining robust performance in rare event prediction. Its success can be attributed to its ability to maintain topological relationships among embeddings while deeply integrating molecular network and interaction data, enabling strong predictive capabilities across balanced and imbalanced scenarios.

**Fig. 1:**
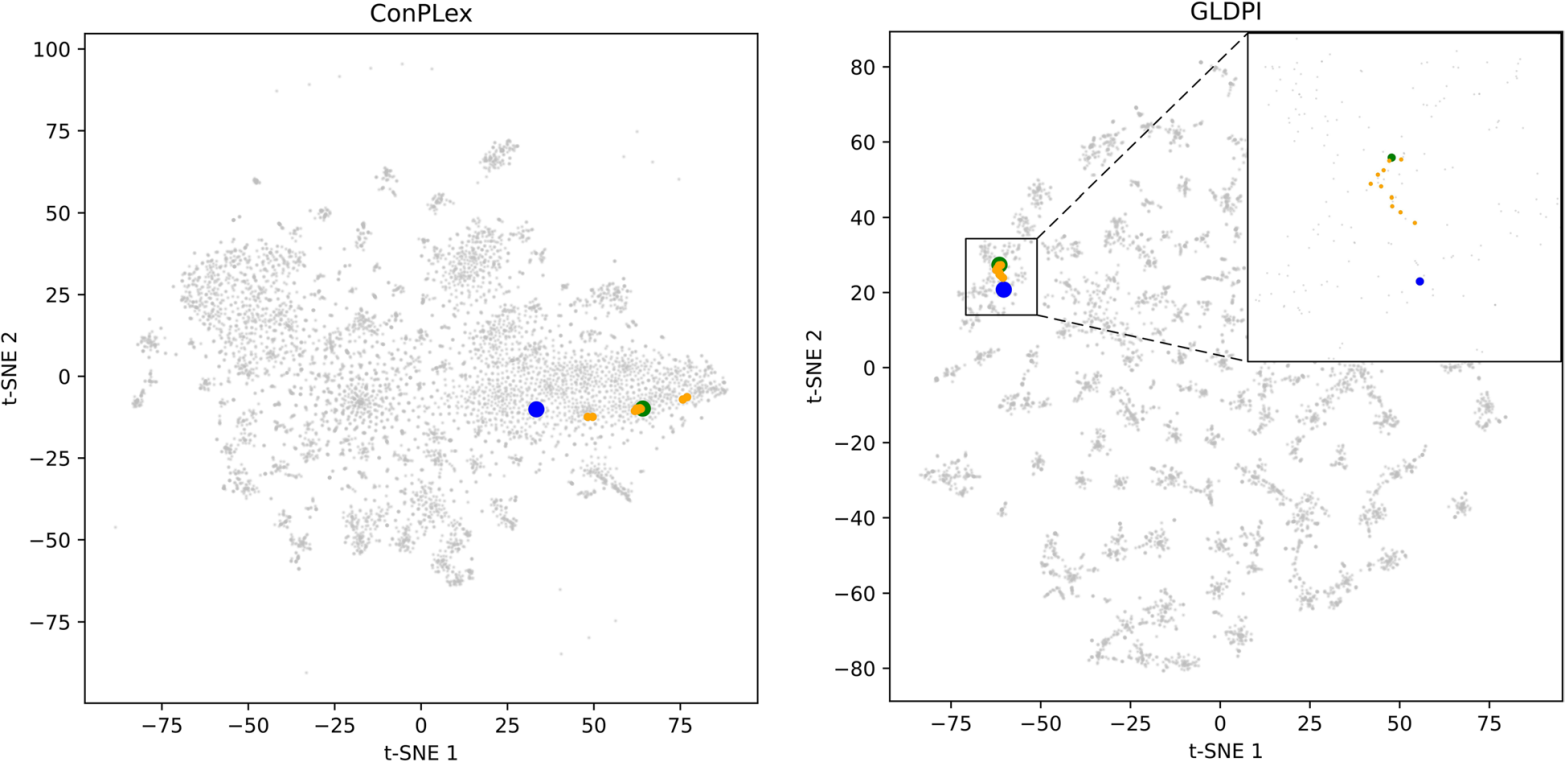
The t-SNE visualizations of drug and protein molecules in the shared embedding space. In the figure, blue dots represent the drug Guanabenz, green dots represent the protein Alpha-2A adrenergic receptors, yellow dots represent the ten proteins most similar to Alpha-2A adrenergic receptors, and gray dots represent other drugs and proteins

It is worth noting that under severely imbalanced conditions, ConPLex also outperforms other baseline methods. This is because its shared drug-protein embedding space setup partially preserves the topological relationships between molecular embeddings. As a result, proteins similar to those associated with a particular drug are more likely to form links with it. As shown in Fig. 1, the drug Guanabenz interacts with the protein Alpha-2A adrenergic receptor, and the 10 proteins most similar to the Alpha-2A adrenergic receptor are also located near Guanabenz in ConPLex’s shared embedding space. Consequently, they achieve higher predictive scores for interaction with Guanabenz. However, compared to GLDPI, ConPLex lacks alignment with the structural information of known drug-protein networks, leaving it more susceptible to interference from unrelated drugs and proteins. This finding further highlights the critical importance of maintaining topological relationships between embeddings for drug-protein interaction prediction in imbalanced scenarios.

To more clearly demonstrate the ability of GLDPI to effectively identify positive samples in highly imbalanced datasets, we evaluated its performance by considering the number of positive samples among the Top-k predicted results. The test sets for both the BindingDB and Biosnap datasets were constructed with a positive-to-negative ratio of 1:1000, as this ratio is closer to real-world scenarios [25]. A higher number of positive samples among the Top-k predicted results indicates the model’s more vital predictive ability.

**Fig. 2:**
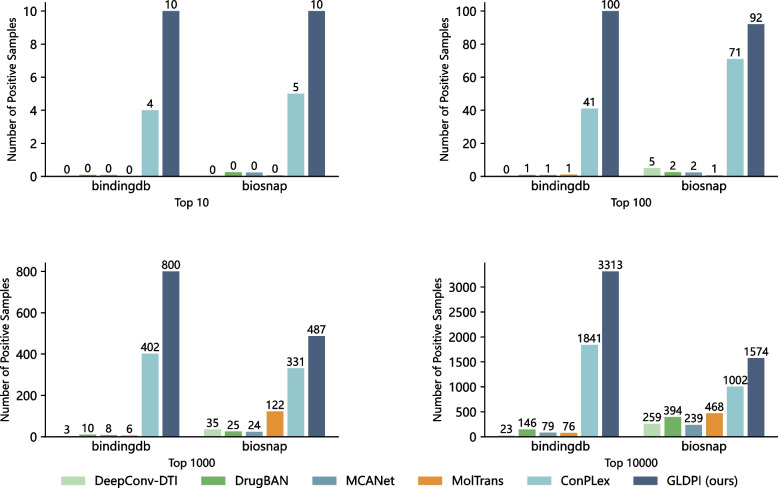
The number of positive samples in the top 10, 100, 1000, and 10,000 predicted candidates by GLDPI and the baselines, respectively, when the imbalance ratio is 1:1000

As shown in Fig. 2, in all four scenarios, the results are similar, with GLDPI significantly outperforming ConPLex. The other four methods can only identify a few positive samples. Furthermore, as we focus on samples with higher rankings, the lead of GLDPI over other baselines becomes more pronounced. The results highlight GLDPI’s robustness in handling imbalanced datasets and its ability to prioritize biologically meaningful interactions at higher rankings, which is crucial for experimental validation. Furthermore, GLDPI’s effectiveness in identifying more positive samples in larger prediction pools indicates its strong generalization and recall capabilities. These attributes make GLDPI a particularly valuable tool for drug discovery pipelines, as it can provide reliable candidate drug-protein pairs for further biological experiments while reducing the effort required to sift through false positives.

### Cold start experiment

Previous experiments have shown that GLDPI can learn more comprehensive knowledge of drug-protein interactions, thereby improving the accuracy of DPI prediction. To further illustrate this, we conducted additional cold-start experiments on the balanced BioSNAP and BindingDB datasets to study the model’s performance on unseen drugs/proteins and independent test datasets.

**Fig. 3:**
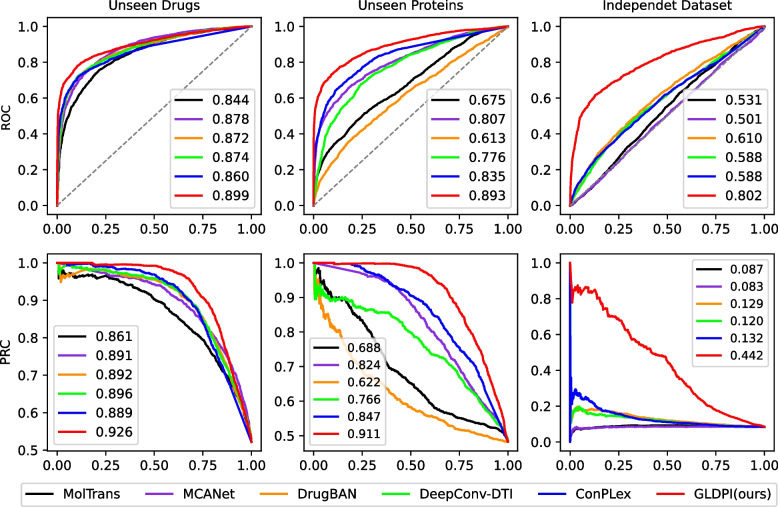
The performance of models on ROC curve (AUROC) and PR curve (AUPR) under different cold start settings

In the BioSNAP dataset, we randomly selected 20% of drugs/proteins, using all drug-protein pairs related to these drugs/proteins (70% as the test set and 30% as the validation set) to evaluate the model’s predictive performance, with the remaining used for model training. This is how we do the unseen drug and unseen protein settings. Compared to predicting DPI for unknown drugs, predicting DPI for unknown proteins is more challenging due to the greater complexity and diversity involved in protein-based predictions. To further increase the difficulty of the prediction task, we trained models on the BindingDB dataset and evaluated their generalization performance using the Davis dataset [33] as an independent test set. The Davis dataset contains dissociation constants for multiple kinase protein families, involving 68 drugs and 379 proteins, with 25,772 drug-protein pairs. Following previous studies [9, 11], we transformed the Davis dataset into a binary dataset. For evaluation, we randomly split the Davis dataset into validation and test sets at a 1:9 ratio. As shown in Fig. 3, the improvement in the predictive performance of GLDPI compared to baseline methods is highly significant. In all three unseen settings, GLDPI achieved the best performance on both AUROC and AUPR metrics. In the more challenging unseen protein settings, GLDPI’s lead was even more remarkable compared to the unseen drug settings. In the most difficult independent dataset, GLDPI outperformed the second-best method by over 30% on both AUROC and AUPR. This suggests that GLDPI, which takes into account network-level information, can better capture relevant information at the molecular level between proteins and thus improve the accuracy of drug-protein interaction prediction.

To further demonstrate the scalability of GLDPI, we applied the GLDPI model, which was trained on the aforementioned BindingDB [32], to predict interactions within the entire BindingDB database [31] using a single 3090 GPU. This database encompasses 1,301,732 drug molecules and 9499 protein molecules, amounting to approximately $1.2 \times 10^{10}$ drug-protein pairs. Remarkably, the entire inference process, including the time for embedding, was completed in under 10 h. This underscores the significant potential of our method to effectively predict drug-protein interactions at a scale comparable to that of the human genome. The high efficiency stems from the fact that, unlike methods such as MolTrans, DeepConv-DTI, MCANet, and DrugBAN, which require extensive computation for each drug-target pair and have quadratic time complexity, GLDPI, similar to ConPLex, has linear time complexity. Once the embeddings are obtained, interactions can be efficiently computed based on distance.

### Ablation experiment

To demonstrate that the outstanding performance of GLDPI stems from maintaining the topological relationships between embeddings, we removed the first two components of GBA_Loss in GLDPI, keeping only the interaction loss function for label alignment. This resulted in a variant called GLDPI (OI_loss). We evaluated the predictive performance of the models using six metrics: AUROC, AUPR, Accuracy, F1-Score (Tables 5, 6, 7, and 8). The experimental results demonstrate that GLDPI (OI_loss) exhibits a performance decline across all seven datasets compared to the full GLDPI model, particularly in AUPR and F1-Score on highly imbalanced datasets. This indicates that the network-level knowledge captured by GBA_Loss is critical for drug-protein interaction prediction, as it significantly improves the model’s ability to predict rare events (i.e., imbalanced classes) by maintaining the topological relationships between embeddings.

**Table 5:** The AUROC performance of GLDPI, ConPlex, and their variants on different datasets

Dataset	GLDPI	GLDPI(OI_loss)	ConPlex	ConPlex(GBA_Loss)
BioSNAP (1:1)	**0.958**	0.941	0.891	0.936
BioSNAP (1:1000)	**0.957**	0.939	0.892	0.935
BindingDB (1:1)	**0.970**	0.968	0.888	0.942
BindingDB (1:1000)	**0.968**	0.887	0.852	0.934
Unseen Drugs	**0.899**	0.899	0.860	0.861
Unseen Proteins	**0.893**	0.882	0.835	0.868
Independent Dataset	**0.802**	0.735	0.588	0.723

**Table 6:** The AUPR performance of GLDPI, ConPlex, and their variants on different datasets

Dataset	GLDPI	GLDPI(OI_loss)	ConPlex	ConPlex(GBA_Loss)
BioSNAP (1:1)	**0.968**	0.949	0.911	0.948
BioSNAP (1:1000)	**0.274**	0.139	0.123	0.181
BindingDB (1:1)	**0.961**	0.961	0.871	0.918
BindingDB (1:1000)	**0.557**	0.249	0.177	0.209
Unseen Drugs	**0.926**	0.919	0.889	0.899
Unseen Proteins	**0.911**	0.894	0.847	0.879
Independent Dataset	**0.442**	0.299	0.132	0.251

**Table 7:** The Accuracy performance of GLDPI, ConPlex, and their variants on different datasets

Dataset	GLDPI	GLDPI(OI_loss)	ConPlex	ConPlex(GBA_Loss)
BioSNAP (1:1)	**0.910**	0.873	0.829	0.882
BioSNAP (1:1000)	**0.999**	0.998	0.998	0.998
BindingDB (1:1)	**0.919**	0.908	0.839	0.873
BindingDB (1:1000)	**0.999**	0.999	0.998	0.999
Unseen Drugs	**0.832**	0.820	0.796	0.803
Unseen Proteins	**0.821**	0.803	0.752	0.776
Independent Dataset	**0.912**	0.872	0.813	0.863

**Table 8:** The F1_score performance of GLDPI, ConPlex, and their variants on different datasets

Dataset	GLDPI	GLDPI(OI_loss)	ConPlex	ConPlex(GBA_Loss)
BioSNAP (1:1)	**0.910**	0.871	0.822	0.878
BioSNAP (1:1000)	**0.330**	0.219	0.200	0.273
BindingDB (1:1)	**0.902**	0.891	0.804	0.842
BindingDB (1:1000)	**0.557**	0.372	0.314	0.329
Unseen Drugs	**0.835**	0.824	0.795	0.800
Unseen Proteins	**0.807**	0.791	0.762	0.785
Independent Dataset	**0.483**	0.325	0.195	0.330

To further illustrate the effectiveness of preserving topological relationships between embeddings in improving predictive model performance, we constructed a single-layer network framework identical to ConPLex and trained it using GBA_Loss as the loss function. The results indicate that although its performance was not as good as GLDPI, the predictive performance of ConPLex (GBA_Loss) was significantly improved compared to the original ConPLex. This demonstrates that for a well-trained ConPLex, incorporating the network-level knowledge of GBA_Loss can still enhance its predictive performance. It also shows that even within other prediction frameworks, GBA_Loss can improve model performance by leveraging network-level knowledge, thereby proving that maintaining topological relationships between embeddings has strong generalizability and potential for broader applications.

The results under the cold-start scenarios, including Unseen Drugs, Unseen Proteins, and the Independent Dataset setting, further validate the importance of topological relationships. GLDPI (OI_loss) showed significantly lower AUPR metrics in these scenarios compared to the complete GLDPI, while ConPLex (GBA_Loss) likewise outperformed the original ConPLex. This indicates that under cold-start conditions, maintaining the topological relationships between embeddings helps enhance the model’s generalization ability. In addition, we performed an ablation study on the drug and protein feature encoding methods. Specifically, for drug encoding, we replaced Morgan fingerprints with Mol2vec [34] and Char2num [11]; for protein encoding, we substituted ESM2 with ProtBert [35] and Char2num [11]. The prediction performance in terms of AUROC, AUPR, Accuracy, and F1_score on the BindingDB and BioSNAP datasets is presented in Additional file 1: Tables S3 and S4. The results demonstrate that Morgan fingerprints and ESM2 consistently provide strong and robust performance. Nevertheless, even when using suboptimal drug and protein encoders instead of the best-performing ones, our method still outperforms the baseline models. This further highlights the robustness and superiority of our framework.

### Case study

**Fig. 4:**
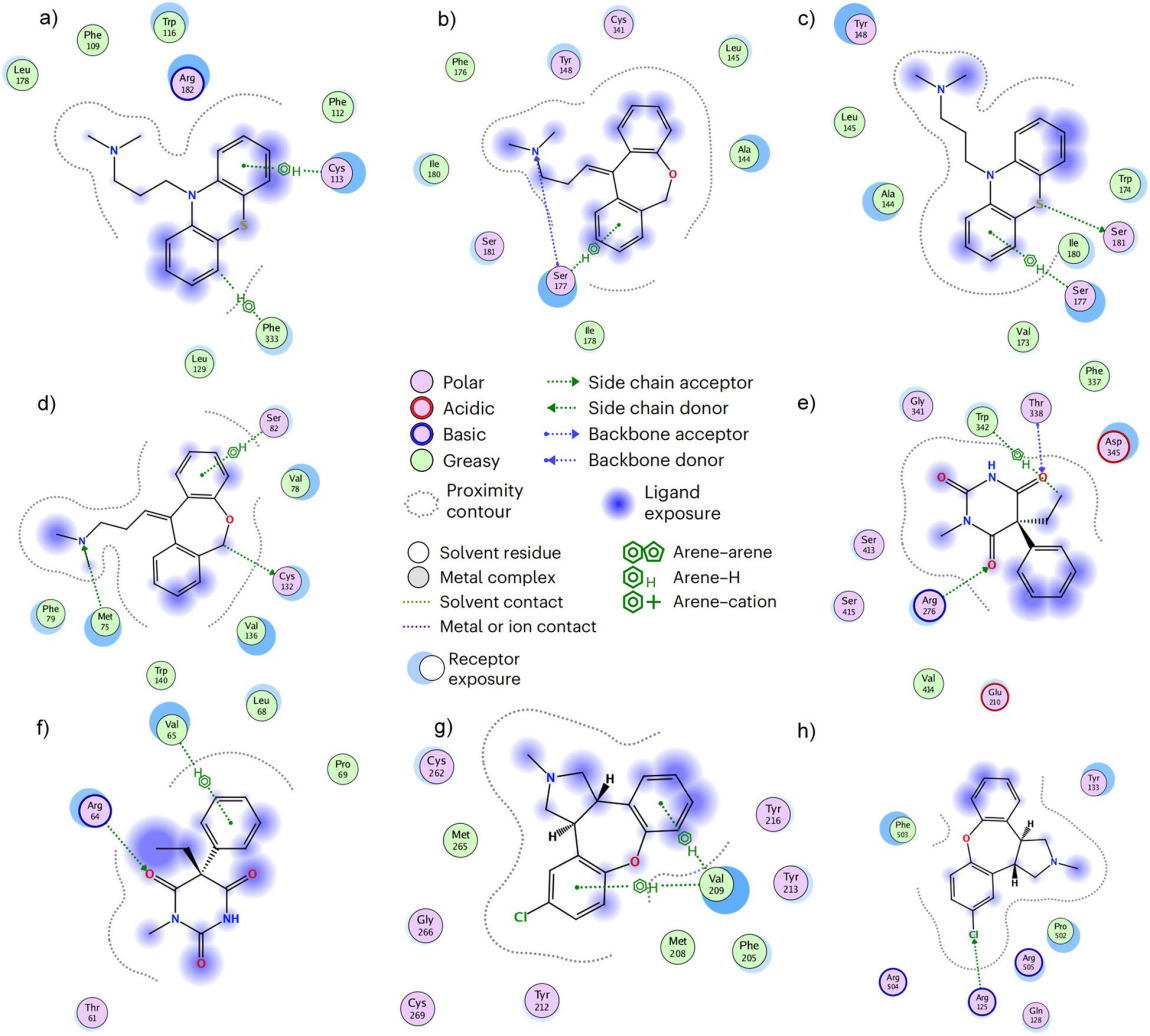
Molecular docking of potential drug-protein pairs predicted by GLDPI. **a** Promazine and DRD5, **b** doxepin and HTR1B, **c** promazine and HTR1B, **d** doxepin and DRD5, **e** methylphenobarbital and GABRB2, **f** methylphenobarbital and GABRE, **g** asenapine and HTR4 **h** asenapine and ADRA1D

To validate the effectiveness of GLDPI in real-world scenarios, we conducted case studies on four drugs (Promazine, Doxepin, Asenapine, and Methylphenobarbital). We used the GLDPI model to predict which new proteins these drugs might interact with. After excluding known interacting proteins, we listed the top 5 proteins for each drug based on their prediction scores in Table 9. Subsequently, we validated the prediction results using the drug-protein interaction records from the DrugCentral database [36]. DrugCentral is an open-access online database, that provides detailed information on approved drugs, including active ingredients, indications, and action mechanisms. Among the 20 predicted targets, 11 were confirmed to interact with the corresponding drugs according to DrugCentral. For 8 of these interactions, binding affinities were available and are reported in Table 9, while the remaining 3 were annotated as interacting pairs without detailed affinity data. For the 9 unverified drug-protein pairs, we further employed ColabFold [37] to predict the 3D structures of the proteins and performed docking simulations. Notably, 8 of the 9 pairs demonstrated favorable docking results through hydrogen bonding or van der Waals interactions as illustrated in Fig. 4, except the Methylphenobarbital-GABRQ pair, which did not exhibit significant binding. These potential interactions show significant promise for the treatment of epilepsy, neurodegenerative diseases, and the alleviation of urinary symptoms. Further investigation into their mechanisms of action and optimization of drug structures could pave the way for the development of more effective and safer therapeutic options, offering patients a wider range of treatment choices. This indicates that GLDPI can discover potential drug-protein interactions in real-world scenarios. As a result, it can significantly shorten the scope of experiments that biologists have to perform when exploring new drug indications.

**Table 9:** Candidate proteins predicted by GLDPI

Drug	Rank	Protein	Prediction score	Interaction	Binding affinity
Promazine	1	HTR2B	0.891	Ture	Ki: 6.66
	2	HTR1A	0.881	Ture	Ki: 5.53
	3	HTR1B	0.880	False	-
	4	DRD5	0.880	False	-
	5	ADRA2B	0.877	Ture	Ki: 6.66
Doxepin	1	HTR1B	0.848	False	-
	2	HTR7	0.839	Ture	-
	3	DRD1	0.833	Ture	Ec50: 6.50
	4	DRD5	0.826	False	-
	5	DRD3	0.822	Ture	Ki: 6.33
Asenapine	1	ADRA1B	0.892	Ture	Ki: 8.41
	2	ADRA1D	0.882	False	-
	3	DRD5	0.860	Ture	Ki: 7.64
	4	HTR4	0.857	False	-
	5	CHRM5	0.843	Ture	Ki: 6.57
Methylphenobarbital	1	GABRB3	0.869	Ture	-
	2	GABRQ	0.868	False	-
	3	GABRB2	0.864	False	-
	4	GABRE	0.861	False	-
	5	GABRG2	0.860	Ture	-

## Conclusions

This paper introduces a highly generalizable and scalable model, GLDPI, which enhances the prediction of drug-protein interactions in imbalanced scenarios by maintaining topological relationships among embeddings. By integrating molecular networks and interaction structure information, GLDPI leverages dedicated drug and protein encoders, along with a heterogeneous drug-protein network, to capture both topological and structural features. During this process, a prior loss function based on the “guilt-by-association” principle aligns embedding topologies with network relationships, enabling the model to achieve substantial improvements in predictive accuracy on imbalanced datasets. Extensive experiments demonstrate that GLDPI achieves significant improvements in predictive accuracy compared to baseline methods on two highly imbalanced benchmark datasets. Moreover, the model exhibits exceptional performance in cold-start scenarios. Beyond drug-protein interaction prediction, our approach can be extended to other entity association prediction tasks, such as drug-disease and microRNA-disease association predictions.

## Methods

**Fig. 5:**
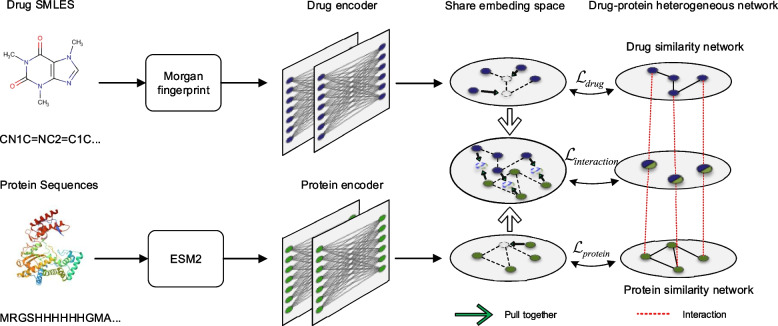
Model architecture of GLDPI. Drug and protein sequences are encoded by the Morgan fingerprint and ESM2 model, then the embeddings are encoded into a shared embedding space using multi-layer neural networks. Meanwhile, the consistency of the topological structure among molecular embeddings and the topological structure of the drug-protein heterogeneous network is maintained by GBA_Loss. GBA_Loss consists of three terms: $\mathcal {L}_{drug}$ ensures the topological consistency between the drug embedding space and the drug similarity network, $\mathcal {L}_{protein}$ enforces topological alignment between the protein embedding space and the protein similarity network, and $\mathcal {L}_{interaction}$ aligns the drug-protein co-embeddings in the shared space with known drug-protein interactions

In this section, we present the GLDPI method, highlighting its motivation and core principles. The model consists of two main components: a deep neural network-based prediction framework and a prior loss function designed for maintaining original topological relationships among embeddings. The model architecture of GLDPI is shown in Fig. 5. The prediction framework learns latent representations of drugs and proteins, using the cosine distance to quantify the likelihood of interactions, with a smaller distance indicating higher interaction likelihood. The prior loss function, termed the guilt-by-association loss function (GBA_Loss), enforces topological relationships through three key constraints that regulate distances between drugs, proteins, and drug-protein pairs in the embedding space. These constraints guide the optimization process to align with the guilt-by-association principle, effectively regularizing the model, maintaining network structure, and reducing overfitting.

### Problem definition

The purpose of the DPI prediction task is to determine whether a drug-protein pair can interact. For a set of *m* drugs represented as $D_c = \{d_1, d_2, ..., d_m\}$, where the drug features are their Simplified Molecular Input Line Entry System (SMILES) representations, and a set of *n* proteins represented as $P_c = \{p_1, p_2, ..., p_n\}$, where the protein features are their amino acid sequences. The interaction status between drug $d_i$ and protein $p_j$ is denoted as $y_{i,j}$. If they can interact, $y_{i,j} = 1$; otherwise, it is 0.

### The prediction framework of GLDPI

Starting with drug SMILES strings and protein amino acid sequences, we encode drugs as Morgan fingerprints [38] and represent proteins using the pre-trained ESM2 protein language model [39], obtaining their initial feature vectors $D\in \mathbb {R}^{d_m}$ and $P \in \mathbb {R}^{d_t}$. These representations are then embedded into a shared embedding space, where interaction likelihood is evaluated, as detailed in the following process.

Given a drug fingerprint $D^{0}\in \mathbb {R}^{d_m}$, we use a two-layer fully connected network to convert it to a latent embedding $D^* \in \mathbb {R}^{d_s}$. In the hidden layer, the embedding vector has a dimension of $d_h$, and the transformation follows:

1 $$\begin{aligned} D^{1} = ReLU\left( \left(BN(W_d^{0}D^{0}+b_d^{0}\right)\right) \end{aligned}$$

2 $$\begin{aligned} D^*=Tanh\left( W_d^{1}D^{1}+b_d^{1}\right) , \end{aligned}$$

where $W_d^{0}\in \mathbb {R}^{d_h\times d_m}$ and $W_d^{1}\in \mathbb {R}^{d_s\times d_h}$ are learnable weight matrices, while $b_d^{0}\in \mathbb {R}^{d_h}$ and $b_d^{1}\in \mathbb {R}^{d_s}$ are learnable bias, Here, $BN(\cdot )$ denotes Batch Normalization, $ReLU(\cdot )$ and Tanh $(\cdot )$ are activation functions.

Given a protein feature $P^{0} \in \mathbb {R}^{d_t}$, we utilize another similar two-layer fully connected network to convert it to a latent embedding $P^* \in \mathbb {R}^{d_s}$. Again, the embedding vector in the hidden layer has a dimension of $d_h$:

3 $$\begin{aligned} P^{1} = ReLU\left( \left(BN(W_p^{0}P^{0}+b_p^{0}\right)\right) \end{aligned}$$

4 $$\begin{aligned} P^*=Tanh\left( W_p^{1}P^{1}+b_p^{1}\right) , \end{aligned}$$

where $W_p^{0}\in \mathbb {R}^{d_h\times d_t}$ and $W_p^{1}\in \mathbb {R}^{d_s\times d_h}$ are learnable weight matrices, $b_p^{0}\in \mathbb {R}^{d_h}$ and $b_p^{1}\in \mathbb {R}^{d_s}$ are learnable bias.

Once we obtain the latent embeddings $D^*$ and $P^*$, the interaction likelihood denoted as $\hat{I}(D^*, P^*)$ is computed as:

5 $$\begin{aligned} \hat{I}(D^*, P^*)= 1-\Phi (D^*, P^*) = \frac{D^*\cdot P^*}{||D^*|| ~ ||P^*||} , \end{aligned}$$

where $\cdot$ denotes the dot product, and $||\cdot ||$ represents the magnitude of a vector, $\Phi (D^*, P^*)$ is the cosine distance between $D^*$ and $P^*$, a smaller cosine distance indicates a higher interaction likelihood.

### Relative distance and GBA_Loss

In the prediction framework, drug-protein interaction likelihood is measured by the cosine distance between their representations in a shared embedding space. The loss function aligns the cosine similarity of molecular embeddings with interaction labels while preserving similarity relationships among drugs or proteins to reinforce the likelihood that similar drugs interact with the same protein, consistent with the guilt-by-association principle.

To implement this, we define relative distances between molecules and design GBA_Loss to maintain topological relationships within the embedding space. GBA_Loss ensures that cosine distances in the embedding space correspond to relative distances derived from the drug-protein heterogeneous network, which are based on drug chemical similarity, protein sequence similarity, and interaction labels. Unlike raw similarity scores, relative distances address inconsistencies in scale between drug and protein similarity distributions. By transforming raw similarity and interaction data into relative distances, we harmonize relationships in the embedding space, aligning molecular representations across scales.

#### Relative distance construction

**Relative distance between drugs:** We compute drug similarities using Morgan fingerprints [38] and Dice similarity [40], resulting in a similarity matrix $S_r$. The relative distance between drugs $d_{i_1}$ and $d_{i_2}$ is defined as:

6 $$\begin{aligned} RD(d_{i_1}, d_{i_2})=1-S_r(d_{i_1}, d_{i_2}). \end{aligned}$$

**Relative Distance Between Proteins:** Protein similarities are calculated using the Smith-Waterman algorithm [41], yielding a similarity matrix $S_p$. Due to the sparsity and small magnitude of Smith-Waterman scores, we perform transformations to make protein similarities comparable with drug similarities. Specifically, for each protein, we sort its similarity scores with other proteins, replace the original scores with the reciprocal of their rank order, and apply a *t-*th root transformation to obtain a new similarity matrix $S_p'$. Transformation details are shown in Algorithm 1, which preserves the relative magnitude of similarities while addressing sparsity. The relative distance between proteins $p_{j_1}$ and $p_{j_2}$ is then:

7 $$\begin{aligned} RD(p_{j_1}, p_{j_2})=1-S_p'(p_{j_1}, p_{j_2}). \end{aligned}$$

**Relative distance between drug and protein:** To ensure interacting drug-protein pairs have smaller distances in the embedding space, relative distances are defined based on interaction labels $y_{i,j}$:

8 $$\begin{aligned} RD(d_i, p_j)=1-y_{i,j}. \end{aligned}$$

**Algorithm 1:**
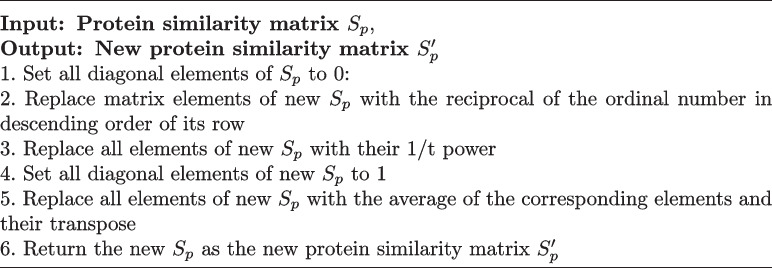
$$S_p'=transform(S_p)$$

#### GBA_Loss

In this section, we construct a drug-protein heterogeneous network based on drug-relative distances, protein-relative distances, and drug-protein relative distances. Then, we propose the GBA_Loss function, which incorporates the guilt-by-association principle approach to guide the representation learning of drugs and proteins. GBA_Loss ensures that, after complex embedding transformations, the molecular representations of drugs and proteins maintain topological relationships consistent with the drug-protein heterogeneous network. This enables the model to learn at both the feature interaction and DPI network levels, allowing for comprehensive knowledge of DPIs and improving predictive accuracy in severely imbalanced scenarios.

The GBA_Loss consists of three parts. The first part $\mathcal {L}_{drug}$is responsible for aligning the distance between drug representations in the shared embedding space with their corresponding relative distance in the drug network. The second part $\mathcal {L}_{protein}$ is responsible for aligning the distance between protein representations in the shared embedding space with their corresponding relative distance in the protein network. The third part $\mathcal {L}_{interaction}$ is responsible for aligning the distance between drug and protein representations in the shared embedding space with the relative distance between the corresponding drug-protein pairs. The GBA_Loss is defined as:

9 $$\begin{aligned} GBA\_Loss= & \mathcal {L}_{drug} + \mathcal {L}_{protein} + \mathcal {L}_{interaction} \nonumber \\= & \sum \limits _{i_1,i_2}^m(\Phi (D^*_{i_1}, D^*_{i_2}) - RD(d_{i_1}, d_{i_2}))^2 \nonumber \\ & +\sum \limits _{j_1,j_2}^n(\Phi (P^*_{j_1}, P^*_{j_2}) - RD(p_{j_1}, p_{j_2}))^2 \nonumber \\ & + \lambda \sum \limits _{i,j}(\Phi (D^*_i, P^*_j)- RD(d_i, p_j))^2 , \end{aligned}$$

where $D^*_i(i\in \{1,2,\cdots ,m\})$ and $P^*_j(j\in \{1,2,\cdots ,n\})$ denote the representation of drug $d_i$ and protein $p_j$, respectively, within the shared embedding space, $\Phi (\cdot ,\cdot )$ represents cosine distance between two vectors, $RD(\cdot ,\cdot )$ represents the relative distance of between two molecules and $\lambda$ is a hyperparameter for adjusting the weight.

## Supplementary Information

Additional file 1: Table S1-S4. Supplementary Tables for additional performance results. Table S1-The further prediction performance of GLDPI on the BioSNAP dataset. Table S2-The further prediction performance of GLDP on the BindingDB dataset. Table S3-GLDPI classification results on benchmark datasets using different drug feature generation methods. Table S4-GLDPI classification results on benchmark datasets using different protein feature generation methods.

## Data Availability

The datasets and code supporting the conclusions of this article are available in the Zenodo repository https://zenodo.org/records/15180746. All data generated or analysed during this study are included in this published article, its supplementary information files and publicly available repositories. The BindingDB dataset can be accessed at https://www.bindingdb.org/rwd/bind/index.jsp; the Biosnap dataset can be obtained at https://github.com/samsledje/ConPLex dev/tree/main/dataset/BIOSNAP; the Davis dataset can be found at https://github.com/hkmztrk/DeepDTA/tree/master/data/davis.

## Abbreviations

DPI: Drug-protein interaction
SMILES: Simplified Molecular Input Line Entry System
AUROC: Area under receiver operating characteristic curve
AUPR: Area under the precision-recall curve
GBA_Loss: Guilt-by-association loss function
