# Supplementary material for: Accurate prediction of drug-protein interactions by maintaining the original topological relationships among embeddings
Source: BMC Biol. 2025 Aug 5;23:243. doi: 10.1186/s12915-025-02338-0 (PMC12326672; doi:10.1186/s12915-025-02338-0)
Supplement: Supplementary file 1 — Additional file 1: Table S1-S4. Supplementary Tables for additional performance results. Table S1-The further prediction performance of GLDPI on the BioSNAP dataset. Table S2-The further prediction performance of GLDP on the BindingDB dataset. Table S3-GLDPI classification results on benchmark datasets using different drug feature generation methods. Table S4-GLDPI classification results on benchmark datasets using different protein feature generation methods. [file 12915_2025_2338_MOESM1_ESM.docx]

Additional file 1

**Accurate prediction of drug-protein interactions by maintaining the original topological relationships among embeddings**

We conducted five randomized experiments using seeds [20, 40, 60, 80, 100], recording the performance of GLDPI across various evaluation metrics as well as the standard deviation of the results. As shown in Tables S1 and S2, the GLDPI model demonstrates strong overall performance on both datasets, achieving high scores across all four metrics—AUROC, AUPR, Accuracy, and F1-score—indicating robust classification capability and stability. Moreover, the consistently low standard deviations across different negative sample ratios reflect the model's high consistency and reproducibility across multiple independent runs.

**Table S1.** The further prediction performance of GLDPI on the BioSNAP dataset.

| **Random seed** | **Ratio** | **AUROC** | **AUPR** | **Accarury** | **F1_score** |
| --- | --- | --- | --- | --- | --- |
| 20 | 1:1 | 0.9583 | 0.9684 | 0.9104 | 0.9082 |
|  | 1:10 | 0.9575 | 0.8641 | 0.9670 | 0.8125 |
|  | 1:100 | 0.9573 | 0.6323 | 0.9926 | 0.6265 |
|  | 1:1000 | 0.9573 | 0.2787 | 0.9985 | 0.3339 |
| 40 | 1:1 | 0.9584 | 0.9681 | 0.9115 | 0.9093 |
|  | 1:10 | 0.9575 | 0.8628 | 0.9671 | 0.8131 |
|  | 1:100 | 0.9574 | 0.6306 | 0.9929 | 0.6265 |
|  | 1:1000 | 0.9573 | 0.2765 | 0.9985 | 0.3309 |
| 60 | 1:1 | 0.9584 | 0.9674 | 0.9074 | 0.9051 |
|  | 1:10 | 0.9575 | 0.8559 | 0.9663 | 0.8045 |
|  | 1:100 | 0.9572 | 0.6133 | 0.9926 | 0.6069 |
|  | 1:1000 | 0.9572 | 0.2614 | 0.9984 | 0.3178 |
| 80 | 1:1 | 0.9585 | 0.9674 | 0.9074 | 0.9051 |
|  | 1:10 | 0.9575 | 0.8559 | 0.9663 | 0.8045 |
|  | 1:100 | 0.9572 | 0.6133 | 0.9926 | 0.6069 |
|  | 1:1000 | 0.9572 | 0.2614 | 0.9984 | 0.3178 |
| 100 | 1:1 | 0.9571 | 0.9684 | 0.9128 | 0.9084 |
|  | 1:10 | 0.9576 | 0.8634 | 0.9672 | 0.8120 |
|  | 1:100 | 0.9575 | 0.6330 | 0.9924 | 0.6241 |
|  | 1:1000 | 0.9574 | 0.2801 | 0.9991 | 0.3357 |
| Average | 1:1 | 0.9581±0.0004 | 0.9680±0.0004 | 0.9076±0.0016 | 0.9103±0.0021 |
|  | 1:10 | 0.9572±0.0006 | 0.8615±0.0033 | 0.9669±0.0004 | 0.8105±0.0035 |
|  | 1:100 | 0.9571±0.0006 | 0.6272±0.0081 | 0.9926±0.0002 | 0.6198±0.0076 |
|  | 1:1000 | 0.9571±0.0005 | 0.2741±0.0075 | 0.9986±0.0003 | 0.3299±0.0070 |

Experiments were conducted with five random repetitions with seed [20,40,60,80,100].

**Table S2.** The further prediction performance of GLDPI on the BindingDB dataset.

| **Random seed** | **Ratio** | **AUROC** | **AUPR** | **Accarury** | **F1_score** |
| --- | --- | --- | --- | --- | --- |
| 20 | 1:1 | 0.9700 | 0.9614 | 0.9191 | 0.9027 |
|  | 1:10 | 0.9681 | 0.9195 | 0.9801 | 0.8867 |
|  | 1:100 | 0.9677 | 0.8288 | 0.9963 | 0.8120 |
|  | 1:1000 | 0.9677 | 0.5594 | 0.9990 | 0.5579 |
| 40 | 1:1 | 0.9693 | 0.9601 | 0.9191 | 0.9017 |
|  | 1:10 | 0.9681 | 0.9188 | 0.9801 | 0.8863 |
|  | 1:100 | 0.9676 | 0.8263 | 0.9963 | 0.8131 |
|  | 1:1000 | 0.9676 | 0.5555 | 0.9990 | 0.5553 |
| 60 | 1:1 | 0.9699 | 0.9611 | 0.9195 | 0.9031 |
|  | 1:10 | 0.9673 | 0.9191 | 0.9799 | 0.8874 |
|  | 1:100 | 0.9668 | 0.8293 | 0.9963 | 0.8124 |
|  | 1:1000 | 0.9668 | 0.5619 | 0.9990 | 0.5600 |
| 80 | 1:1 | 0.9701 | 0.9613 | 0.9190 | 0.9025 |
|  | 1:10 | 0.9685 | 0.9195 | 0.9800 | 0.8857 |
|  | 1:100 | 0.9681 | 0.8273 | 0.9963 | 0.8127 |
|  | 1:1000 | 0.9681 | 0.5562 | 0.9990 | 0.5554 |
| 100 | 1:1 | 0.9703 | 0.9611 | 0.9184 | 0.9007 |
|  | 1:10 | 0.9683 | 0.9182 | 0.9800 | 0.8867 |
|  | 1:100 | 0.9680 | 0.8255 | 0.9962 | 0.8112 |
|  | 1:1000 | 0.9680 | 0.5537 | 0.9990 | 0.5539 |
| Average | 1:1 | 0.9699±0.0004 | 0.9611±0.0003 | 0.9190±0.0004 | 0.9092±0.0010 |
|  | 1:10 | 0.9681±0.0004 | 0.9190±0.0005 | 0.9800±0.0001 | 0.8865±0.0006 |
|  | 1:100 | 0.9676±0.0005 | 0.8274±0.0016 | 0.9963±0.0000 | 0.8123±0.0006 |
|  | 1:1000 | 0.9676±0.0005 | 0.5573±0.0033 | 0.9990±0.0000 | 0.5565±0.0024 |

Experiments were conducted with five random repetitions with seed [20,40,60,80,100].

Before selecting the Morgan fingerprint and ESM2 as the default feature extractors for drug and protein representations in GLDPI, we systematically evaluated several alternative encoding methods. For drug encoding, we compared Mol2vec[1], Char2num [2], and Morgan fingerprint [3]; for protein encoding, we assessed Char2num [1], ProtBert [4], and ESM2 [5]. Tables S3 and S4 evaluated their performance across multiple datasets and metrics. Results show that encoding effectiveness can vary by task, and no single method was consistently superior. However, the Morgan fingerprint and ESM2 consistently delivered strong and robust performance, making them the preferred choice for GLDPI. Moreover, the experimental results show that even when suboptimal drug and protein encoders are used instead of the best-performing ones, our method still consistently outperforms baseline models. This further highlights the robustness of our framework.

**Table S3.** GLDPI classification results on benchmark datasets using different drug feature generation methods.

| **Dataset** | **Drug Features** | **AUROC** | **AUPR** | **Accarury** | **F1_score** |
| --- | --- | --- | --- | --- | --- |
| BindingDB(1:1) | Morgan | **0.970** | **0.961** | **0.919** | **0.902** |
|  | Char2num | 0.931 | 0.905 | 0.863 | 0.830 |
|  | Mol2vec | 0.968 | 0.957 | 0.916 | 0.897 |
| BioSNAP (1:1) | Morgan | **0.958** | **0.968** | **0.910** | **0.908** |
|  | Char2num | 0.930 | 0.944 | 0.866 | 0.862 |
|  | Mol2vec | 0.956 | 0.965 | 0.904 | 0.902 |
| Unseen Drug | Morgan | **0.899** | 0.926 | **0.835** | 0.832 |
|  | Char2num | 0.816 | 0.855 | 0.744 | 0.748 |
|  | Mol2vec | **0.899** | **0.928** | 0.833 | **0.835** |
| Unseen Protein | Morgan | **0.893** | 0.911 | 0.807 | **0.821** |
|  | Char2num | 0.847 | 0.877 | 0.786 | 0.770 |
|  | Mol2vec | 0.892 | **0.913** | **0.822** | 0.818 |

**Table S4.** GLDPI classification results on benchmark datasets using different protein feature generation methods.

| **Dataset** | **Protein Features** | **AUROC** | **AUPR** | **Accarury** | **F1_score** |
| --- | --- | --- | --- | --- | --- |
| BindingDB (1:1) | ESM2 | 0.970 | 0.961 | 0.919 | 0.902 |
|  | Protbert | 0.969 | 0.959 | 0.919 | 0.900 |
|  | Char2num | **0.972** | **0.963** | **0.921** | **0.905** |
| BioSNAP (1:1) | ESM2 | **0.958** | **0.968** | 0.910 | **0.908** |
|  | Protbert | 0.955 | 0.967 | **0.911** | 0.907 |
|  | Char2num | 0.947 | 0.959 | 0.890 | 0.885 |
| Unseen Drug | ESM2 | 0.899 | 0.926 | **0.835** | 0.832 |
|  | Protbert | **0.900** | **0.930** | 0.835 | **0.834** |
|  | Char2num | 0.875 | 0.913 | 0.811 | 0.814 |
| Unseen Protein | ESM2 | **0.893** | **0.911** | 0.807 | **0.821** |
|  | Protbert | 0.888 | 0.908 | **0.823** | 0.806 |
|  | Char2num | 0.676 | 0.721 | 0.483 | 0.651 |

1. Jaeger S, Fulle S, Turk S. Mol2vec: unsupervised machine learning approach with chemical intuition. J Chem Inf Model. 2018;58(1):27-35.
2. Ozturk H, Ozgur A, Ozkirimli E. DeepDTA: deep drug-target binding affinity prediction. Bioinformatics. 2018;34(17):i821-i829.
3. Morgan HL. The generation of a unique machine description for chemical structures-a technique developed at chemical abstracts service. J Chem Doc. 1965;5(2):107-113.
4. Elnaggar A, Heinzinger M, Dallago C, Rehawi G, Wang Y, Jones L, et al. Prottrans: Toward understanding the language of life through self-supervised learning. IEEE Trans Pattern Anal Mach Intell. 2021;44(10):7112-7127.
5. Rives A, Meier J, Sercu T, Goyal S, Lin Z, Liu J, et al. Biological structure and function emerge from scaling unsupervised learning to 250 million protein sequences. Proc Natl Acad Sci USA. 2021;118(15):e2016239118.
